# Multi-targeted protection of Yuping Tongqiao against allergic rhinitis: suppression of inflammatory response via TSLP signaling and reinforcement of epithelial barrier integrity via AhR signaling

**DOI:** 10.3389/falgy.2026.1824120

**Published:** 2026-05-08

**Authors:** Zhixin Wang, Jingqi Cai, Jiahao Wu, Haoran Zheng, Min Shi, Yanan Tong, Yunlong Hou, Zhenhua Jia, Hui Qi

**Affiliations:** 1 State Key Laboratory for Innovation and Transformation of Luobing Theory, Shijiazhuang, Hebei, China; 2 Graduate School, Hebei University of Chinese Medicine, Shijiazhuang, Hebei, China; 3 Beijing University of Chinese Medicine School of Chinese Medicine, Beijing, China; 4 Graduate School, Hebei Medical University, Shijiazhuang, China; 5 Shanghai Yiling Pharmaceutical Co., Ltd., Shanghai, China; 6 High-level TCM Key Disciplines of National Administration of Traditional Chinese Medicine – Luobing Theory, Hebei Yiling Hospital, Shijiazhuang, Hebei, China

**Keywords:** AhR, allergic rhinitis, epithelial barrier integrity, TSLP, yuping tongqiao

## Abstract

**Background:**

Allergic rhinitis (AR) is a prevalent chronic inflammatory disorder characterized by persistent inflammation and nasal epithelial barrier disruption. Restoring epithelial integrity and modulating the inflammatory cascade are considered promising therapeutic strategies for AR.

**Purpose:**

This study aims to systematically investigate the therapeutic efficacy of Yuping Tongqiao Tablet (YPTQ), a traditional Chinese medicine formula, in the treatment of AR and to decipher its potential mechanisms and active ingredients focusing on inflammation amelioration and barrier restoration.

**Methods:**

An AR model was established in Sprague-Dawley rats that were sensitized and challenged with OVA. After intervention with different doses of YPTQ, nasal pathological injury and inflammatory cell infiltration were evaluated. *In vitro*, a human nasal epithelial cell (RPMI-2650) injury model was induced using house dust mites (HDM). Following YPTQ treatment, the expression levels of inflammatory factors and barrier-related proteins were assessed. Furthermore, the potential mechanisms and targets of YPTQ and its active monomers against AR were investigated by integrating network pharmacology analysis and molecular docking with *in vitro* experiments.

**Results:**

In the OVA-induced AR rat model, YPTQ effectively alleviated nasal symptoms, reduced histopathological damage and inflammatory cell infiltration, and suppressed overall inflammatory levels. In the HDM-induced RPMI-2650 cell injury model, YPTQ significantly inhibited inflammatory cytokines release and upregulated the tight junction protein ZO-1, thereby enhancing epithelial barrier function. Moreover, integrated analysis combining network pharmacology, molecular docking, and both *in vitro* and *in vivo* validation confirmed that YPTQ and its active ingredient, kaempferol, exert therapeutic effects through two main pathways. Firstly, they down-regulated the expression of TSLP and inhibited the migration of DCs, which subsequently alleviated nasal inflammation. Secondly, they up-regulated and activated the expression of AhR and the downstream CYP1A1, which in turn promoted the expression of a barrier-associated protein, contributing to the restoration of nasal epithelial barrier integrity.

## Introduction

Allergic rhinitis (AR) is a common chronic non-infectious nasal mucosal disease worldwide, affecting approximately 10%–40% of the global population, and its prevalence continues to rise due to environmental changes such as global warming and increased air pollution (1, 2). In the USA, the prevalence of physician-diagnosed allergic rhinitis is about 15%, and patients self-report abnormal nasal symptoms up to 30% of cases (3). In China, an epidemiological survey conducted in six northern Chinese cities reported that the prevalence of AR ranged from 18.6% to 52.9% in 2018 (4). Clinically, AR is characterized by symptoms such as sneezing, rhinorrhea, and nasal congestion, which directly impose a significant health burden that impacts patients' quality of life (e.g., sleep, and comorbidities like asthma) and restricted social activities (1, 2). In addition, the high prevalence of AR imposes a substantial socioeconomic burden, including direct medical costs and indirect productivity losses. In China, the total annual direct and indirect socioeconomic cost of AR reaches as high as 326.8 billion CNY (4). While, in the United States, direct medical costs reach approximately $1.15 billion annually (5). AR symptoms are typically triggered by exposure to aeroallergens such as house dust mites (HDM), pollen, animal dander, and mold spores (6, 7). Other than that, the pathogenesis of AR also involves intricate interactions among genetic susceptibility, environmental allergens, and dysregulated immune responses (7, 8). Current pharmacological interventions for AR, including intranasal corticosteroids (e.g., fluticasone propionate), oral and intranasal antihistamines (e.g., loratadine), and oral leukotriene receptor antagonists (e.g., pranlukast), primarily provide symptomatic relief rather than targeting the underlying disease mechanisms. In addition, these treatments are often associated with adverse effects, high costs, or insufficient efficacy in a subset of patients (9). Therefore, there is an urgent need to develop safer, more effective, and affordable treatment strategies that address the root causes of AR.

The pathophysiology of AR is largely driven by type 2 inflammatory pathways, which are activated when allergens disrupt the nasal epithelial barrier, triggering innate and adaptive immune cascades (10). Central to this process is thymic stromal lymphopoietin (TSLP), an epithelial-derived cytokine that acts as an upstream “gatekeeper” of allergic inflammation. Upon exposure to allergens, pollutants, or pathogens, TSLP expression is significantly elevated in the nasal mucosa of AR patients and correlates with disease severity (11). TSLP, in turn, activates dendritic cells (DCs) to promote the differentiation of naïve T cells into T-helper 2 (Th2) cells, which secrete key cytokines such as IL-4, IL-5, and IL-13. These cytokines facilitate immunoglobulin E (IgE) synthesis, eosinophil infiltration, and mucus hypersecretion, thereby perpetuating allergic inflammation (12). Moreover, genetic polymorphisms in TSLP have been linked to increased AR susceptibility, underscoring its critical role in disease pathogenesis (13). Given its position as an upstream regulator, TSLP represents a promising therapeutic target for interrupting the inflammatory cascade in AR.

Another hallmark of AR is the impairment of the nasal epithelial barrier, which facilitates allergen penetration and immune activation. The aryl hydrocarbon receptor (AhR) pathway, which senses environmental ligands such as dietary metabolites and pollutants, plays a key role in regulating epithelial integrity (14). In AR patients, diminished AhR activity or ligand availability can compromise barrier function by downregulating tight junction proteins, including ZO-1, occluding, and claudins, thereby increasing mucosal permeability (15). Additionally, AhR activation modulates inflammatory responses by suppressing pro-inflammatory cytokines, such as IL-6, TNF-α, and IL-1β, while promoting anti-inflammatory signals like IL-22. It has been shown that AhR deficiency exacerbates colitis through enhancing inflammatory cytokine production, whereas AhR agonists alleviate symptoms by reinforcing barrier function and suppressing Th17 responses (16). These findings suggest that enhancing AhR activity may protect the nasal epithelium in AR by enhancing barrier integrity and mitigating inflammation.

Importantly, a vicious cycle links TSLP overexpression and AhR suppression: environmental pollutants inhibit AhR signaling, disrupting tight junctions and facilitating allergen penetration, which subsequently elevates TSLP overexpression. In turn, TSLP-driven inflammation further compromises barrier function by degrading barrier proteins such as occludin and ZO-1. This interplay, together with the observation that a subset of patients exhibits suboptimal responses to existing therapies, highlights the limitations of single-target therapies and supports a dual-target approach for AR that simultaneously suppresses TSLP-mediated inflammation and enhances AhR-dependent barrier restoration.

Traditional Chinese medicine (TCM) offers a holistic, system-based paradigm for AR management. In TCM theory, AR was classified as “Bi Qiu” (鼻鼽), which is primarily attributed to underlying imbalances such as “lung-spleen qi deficiency”. TCM interventions are increasingly recognized for their potential to deliver comprehensive symptom relief, modulate immune responses, and exhibit favorable safety profiles (17). Yuping Tongqiao (YPTQ, 芪防鼻通片), the first innovative TCM formula approved for registration in Macao, was developed by modifying classical formulas including Yu Ping Feng San (18, 19) and Xin Yi San, with complementary herbs such as galangal. YPTQ is designed specifically to address the TCM pathological mechanism of “lung-spleen dual deficiency”. Evidence from a multicenter, randomized, double-blind, placebo-controlled phase III clinical trial has demonstrated that YPTQ significantly alleviates nasal and ocular symptoms in patients with persistent AR and improves patients' quality of life, with a safety profile comparable to placebo (20). However, the precise molecular mechanisms underlying the therapeutic effects of YPTQ in AR remain incompletely elucidated. The present study systematically investigates the dual regulatory role of YPTQ in modulating TSLP-dependent inflammatory responses and AhR-mediated epithelial barrier protection in the context of AR. Our findings not only decipher the mechanistic basis of YPTQ at a molecular level but also provide an evidence-based foundation for its clinical application. Importantly, this work highlights the novelty of bridging traditional TCM formulae with contemporary immunopharmacological insights, offering a validated model for the integration of traditional medicine and modern scientific discovery.

## Material and methods

### Reagent preparation

#### Preparation of ovalbumin (OVA) sensitization solution

The ovalbumin (OVA) sensitization solution was freshly prepared by dissolving 30 mg of OVA (as the antigen) and 3 g of aluminum hydroxide powder (as the adjuvant) in 100 mL of physiological saline.

#### Preparation of house dust Mite (HDM) extract solution

House dust mites (HDM) extract (XPB82D3A2.5, GREER, USA) was dissolved in 2.5 mL of pure water. The solution was gently mixed and then sterilized by filtration through a 0.22 μm polyethersulfone membrane filter (Millipore, USA). The HDM solution was then diluted with pure water to a final concentration of 2 mg/mL, and stored at −20 °C and used within one year.

#### Preparation of YPTQ tablet solutions

YPTQ tablets, a Chinese herbal formula composed of composed of *Astragalus membranaceus* (Fisch.) Bunge (Huangqi), *Atractylodes macrocephala* Koidz (Baizhu), *Saposhnikovia divaricata* (Turcz.) Schischk (Fangfeng), *Magnolia biondii* Pamp (Xinyi), *Angelica dahurica* (Baizhi), *Alpinia officinarum* Hance (Gaoliangjiang), *Notopterygium incisum* Ting ex H. T. Chang (Qianghuo), *Paeonia suffruticosa* Andr. (Mudanpi), *Cryptotympana atrata* (Fabricius) (Chantui), *Prunus mume* (Sieb.) (Wumei), and *Glycyrrhiza uralensis* Fisch (Gancao) (Supplementary Table S1), were provided by Beijing Yiling Pharmaceutical Co., Ltd. (A2305003, Beijing, China).

For oral gavage administration to rats, YPTQ tablets were ground into a homogeneous powder and dissolved in physiological saline to achieve the desired concentrations. The administered dose for rats was calculated based on the clinical adult human dose of 5.64 g/day (assuming a standard body weight of 60 kg). Using a standard body surface area conversion factor (approximately 8 times the human equivalent dose for rats), the calculated low and high doses for rats were 0.752 g/kg and 1.504 g/kg, respectively.

For cell experiments, the powder was dissolved in Minimum Essential Medium (MEM, Gibco, USA) and stirred magnetically for 1 h before being sonicated for an additional hour to enhance extraction efficiency. Subsequently, the mixture was centrifuged at 8,000 g for 30 min, and the supernatant was collected and sterilized by filtration through a 0.22 μm filter to obtain a stock solution with a concentration of 50 mg/mL, which was aliquoted and stored at −20 °C until use. To determine the appropriate concentration range for YPTQ *in vitro*, a preliminary cytotoxicity assay was performed using the CCK-8 method. The results showed that the maximum non-toxic concentration of YPTQ in RPMI-2650 cells was 800 μg/mL (cell viability >90%, Supplementary Figure S1). In subsequent experiments, we observed that the therapeutic effects of 800 μg/mL YPTQ were comparable to those of 400 μg/mL, and that 400 μg/mL YPTQ already exhibited significantly superior efficacy compared to the positive control drug, loratadine. Therefore, three concentrations (100, 200, and 400 μg/mL) were selected for all subsequent *in vitro* experiments. Immediately prior to cell treatment, working solutions with final concentrations of 100, 200, and 400 μg/mL were freshly prepared by diluting the stock solution with the appropriate cell culture medium.

#### Preparation of kaempferol solution

A stock solution of kaempferol (B21126, Yuanye, China) was prepared in dimethyl sulfoxide (DMSO, Sigma-Aldrich, USA) to prepare a 0.1 M stock solution and stored at −20 °C. Immediately prior to use, working solutions (1, 5, and 10 μM) were freshly prepared by diluting the stock solution with the appropriate cell culture medium.

### Animals

All animal experimental procedures were approved by the Experimental Animal Ethical Committee of Hebei Academy of Integrated Traditional Chinese and Western Medicine (No. N2024085) and conducted in accordance with institutional guidelines. Male Sprague-Dawley (SD) rats (6–8 weeks old, specific pathogen-free, SPF grade) were purchased from Beijing Weitong Lihua Experimental Animal Technology Co., Ltd. (Beijing, China). Rats were acclimatized for one week under standard laboratory conditions prior to experimentation. They were housed in SPF-grade ventilated cages (5 rats per cage) with autoclaved corn cob bedding under controlled environmental conditions: temperature of 22 ± 1 °C, relative humidity of 55 ± 5%, and a 12-h light/dark cycle. Sterilized standard rodent feed and water were provided *ad libitum*.

For surgical procedures, anesthesia was induced and maintained with inhaled isoflurane. Rats were placed in an induction chamber with 3%–4% isoflurane delivered in oxygen until loss of righting reflex. Anesthesia was then maintained via a nose cone with 1.5%–2.5% isoflurane during blood collection. At the end of the experiment, animals were euthanized under deep anesthesia by exsanguination via abdominal aorta blood collection, followed by cervical dislocation to ensure death.

### Establishment of AR rat model and drug administration

After one week of acclimatization, rats were randomly divided into five groups: control group (Control; *n* = 8), AR model group (AR; *n* = 9), low-dose YPTQ group (YPTQ-L, 0.752 g/kg; *n* = 9), high-dose YPTQ group (YPTQ-H, 1.504 g/kg; *n* = 9), and loratadine group (Loratadine, 10 mg/kg; *n* = 9).

The AR model was established following a two-phase protocol, including sensitization phase (Days 1–15) and inspiration phase (Day 16–35). During the sensitization phase, rats received intraperitoneal injections of a sensitizing agent (5 mL/kg) every other day for a total of eight injections. The emulsion contained 30% OVA and 3% aluminum hydroxide dissolved in physiological saline. During the inspiration phase, rats were challenged by bilateral intranasal instillation of 50 μL per side of a 5% OVA solution in physiological saline. For the first 7 days, challenges were performed daily, followed by every other day for the subsequent 14 days. One hour prior to each intranasal challenge, rats in the YPTQ-L, YPTQ-H, and Loratadine groups received the corresponding drug via intragastric administration. Rats in the Control and AR model groups received an equivalent volume of physiological saline. Behavioral symptoms, including sneezing, nasal rubbing (scratching), and rhinorrhea (runny nose), were observed and recorded immediately after each challenge for 30 min by an observer blinded to group allocation. The severity of nasal symptoms was evaluated within 10 min after the final OVA challenge using a scoring system adapted from previous studies (21, 22). In brief, sneezing was scored as follows: 0 = none, 1 = 1–3 sneezes, 2 = 4–10 sneezes, 3 => 10 sneezes; nasal rubbing was scored as follows: 0 = none, 1 = mild rubbing (1–2 times), 2 = moderate rubbing, 3 = vigorous rubbing with facial scratching; rhinorrhea was scored as follows: 0 = none, 1 = mild discharge confined to the nostrils, 2 = moderate discharge extending to the nasal vestibule, 3 = severe discharge covering the face. A total behavioral score of ≥5 points was considered indicative of successful model establishment.

### Induction of AR cellular model (nasal epithelial injury model) and drug treatment

Human nasal epithelial cells (RPMI-2650) were purchased from Jennio Biotechnology Co., Ltd. (Guangzhou, China). Cells were cultured in complete medium containing 89% MEM basal medium (Gibco, USA), 10% fetal bovine serum (FBS, Gibco, USA) and 1% antibiotics (1 × 10^8^ U/L Penicillin, 1 × 10^8^ U/L Streptomycin Sulfate; all from Gibco, USA) at 37 °C with 5% CO₂. The AR cell model was induced by stimulation with 10 μg/mL of HDM extract for 24 h. This concentration was selected based on previous reports (23) and our preliminary experimental results, which showed that 10 μg/mL HDM significantly increased the expression levels of IL-1β and IL-6 while significantly decreasing the expression levels of ZO-1 and Claudin-1 in RPMI-2650 cells (data not shown). For drug intervention studies, cells were divided into following groups: control (Control), model (HDM), YPTQ low-dose (YPTQ-L, HDM + 100 μg/mL YPTQ), medium-dose (YPTQ-M, HDM + 200 μg/mL YPTQ), high-dose (YPTQ-H, HDM + 400 μg/mL YPTQ), and loratadine (HDM + 10^−5^ M Loratadine).

### Collection of nasal lavage fluid (NALF) and cell differential count

Rats were euthanized humanely at the end of the experiment. NALF was collected by gently perfusing 1 mL of ice-cold phosphate-buffered saline (PBS) from the exposed trachea towards the nasopharynx. This procedure was repeated 3 times per side. The pooled NALF was centrifuged at 4 °C, 120 *g* for 10 min. The cell pellet was resuspended in PBS, and differential white blood cell counts were performed using an automated hematology analyzer (Sysmex, Japan).

### Histopathological analysis

To prepare rat nasal tissue samples for histological analysis, the tissues were fixed in 4% paraformaldehyde for 48 h at 4 °C. Subsequently, the fixed tissues were decalcified in a 10% ethylenediaminetetraacetic acid (EDTA) solution (in 0.1 M Tris-HCl buffer, pH 7.4) at 4 °C with constant agitation. Following decalcification, the samples were dehydrated, embedded in paraffin, and sectioned into 5 μm-thick sections for subsequent staining procedures. For histological evaluation, sections were stained with hematoxylin and eosin (H&E, Sigma-Aldrich, USA), toluidine blue (TB, Sigma-Aldrich, USA), and alcian blue (AB, Sigma-Aldrich, USA) kits, respectively, according to the manufacturers' instructions. All stained sections were digitally scanned using a high-resolution pathological slide scanner (NanoZoomer S60, Hamamatsu Photonics, Japan) for subsequent quantitative and qualitative analyses.

### Enzyme-linked immunosorbent assay (ELISA)

Blood samples were centrifuged at 1,500 g, 4 °C for 15 min, and the supernatant was collected. The levels of OVA-sIgE, IFN-*γ*, and IL-4 were measured using commercial ELISA kits (OVA-sIgE: ZCIBIO Technology, Shanghai, China; IFN-*γ* and IL-4: Lianke, Hangzhou, China) according to the manufacturer's instructions.

### Immunofluorescence (IF) staining

For tissue IF staining, nasal paraffin sections were deparaffinized and subjected to antigen retrieval. Sections were then blocked with 3% hydrogen peroxide for 10 min at 37 °C in the dark. Subsequently, the sections were blocked with 5% bovine serum albumin (BSA) for 30 min at room temperature before being incubated with primary antibodies against TSLP (Novus, NBP1-76754, 1:400), ZO-1 (Abcam, ab221547, 1:100), and CYP1A1 (Abcam, ab126887, 1:100) overnight at 4 °C. After washing, sections were incubated with secondary antibodies, either goat anti-rabbit IgG H&L (Alexa Fluor® 647, Abcam, ab150083, 1:500) or donkey anti-rabbit IgG H&L (Alexa Fluor® 488, Abcam, ab150129, 1:500), for 50 min at room temperature in the dark. Cell nuclei were counterstained with 4′,6-diamidino-2-phenylindole (DAPI, Solarbio, Beijing, China).

For cellular IF staining, RPMI-2650 cells were cultured in glass-bottom dishes. After treatment, cells were fixed with 4% paraformaldehyde for 15 min, followed by permeabilization using 0.25% Triton X-100 for 5 min. Subsequently, the cells were blocked with 1% BSA for 1 h at room temperature before being incubated with primary antibodies against TSLP (Abcam, ab47943, 1:100), ZO-1 (Abcam, ab221547, 1:100), or CYP1A1 (Abcam, ab126887, 1:100) overnight at 4 °C. Secondary antibody incubation and DAPI staining were performed as described for tissue sections. All IF images were captured under a Zeiss confocal microscope (Oberkochen, Germany).

### Real-time quantitative polymerase chain reaction (RT-qPCR)

Total RNA was extracted from RPMI-2650 cells using the Eastep® Super Total RNA Extraction Kit (LS1040, Promega Corporation, USA). cDNA was synthesized using the PrimeScript™ RT reagent Kit with gDNA Eraser (RR047A, TaKaRa, Beijing, China). Quantitative PCR was performed using TB Green® Premix Ex Taq™ II (Tli RNaseH Plus) (RR820A, TaKaRa, Beijing, China) on a LightCycler® 96 System (Roche, Germany). The thermal cycling conditions were as follows: initial denaturation at 95 °C for 30 s, followed by 40 cycles of 95 °C for 5 s and 60 °C for 30 s. Glyceraldehyde-3-phosphate dehydrogenase (*GAPDH*) was used as the internal reference gene. The relative mRNA levels of target genes were calculated using the 2^–ΔΔCt^ method (ΔCt value = target gene Ct value − GAPDH gene Ct value). Primer sequences are listed in Table 1.

**Table 1:** Primer sequences used for RT-qPCR.

Gene	Primer Sequence (5′ → 3′)
IL-1β (human)	F:5′-ATGATGGCTTATTACAGTGGCAA-3’
	R:5’-GTCGGAGATTCGTAGCTGGA-3’
IL-6 (human)	F:5’-CACTCACCTCTTCAGAACGAAT-3’
	R:5’-GCAAGTCTCCTCATTGAATCCA-3’
TNF-α (human)	F:5’-CCTCTCTCTAATCAGCCCTCTG-3’
	R:5’-GAGGACCTGGGAGTAGATGAG-3’
IL-4 (human)	F:5’-GCAGTTCCACAGGCACAAG-3’
	R:5’-TCTGGTTGGCTTCCTTCACA-3’
IL-5 (human)	F:5’-TGGAGCTGCCTACGTGTATG-3’
	R:5’-TTCGATGAGTAGAAAGCAGTGC-3’
IL-13 (human)	F:5’-GTATGGAGCATCAACCTGACAG-3’
	R:5’-AGCATCCTCTGGGTCTTCTC-3’
IL-33 (human)	F:5’-GTGACGGTGTTGATGGTAAGAT-3’
	R:5’-AGCTCCACAGAGTGTTCCTTG-3’
TSLP (human)	F:5’-GCTACTCAGGCAATGAAGAAGAGG-3’
	R:5’-CCTTGTAATTGTGACACTTGTTCCAG-3’
ZO-1 (human)	F:5’-CAACATACAGTGACGCTTCACA-3’
	R:5’-CACTATTGACGTTTCCCCACTC-3’
Claudin-1 (human)	F:5’-GTCTTTGACTCCTTGCTGAATCTG-3’
	R:5’-CACCTCATCGTCTTCCAAGCAC-3’
Occludin (human)	F:5’-ATGGCAAAGTGAATGACAAGCGG-3’
	R:5’-CTGTAACGAGGCTGCCTGAAGT-3’
AhR (human)	F:5’-GTCGTCTAAGGTGTCTGCTGGA-3’
	R:5’-CGCAAACAAAGCCAACTGAGGTG-3’
CYP1A1 (human)	F:5’-GATTGAGCACTGTCAGGAGAAGC-3’
	R:5’-ATGAGGCTCCAGGAGATAGCAG-3’
GAPDH (human)	F:5’-GTCAAGGCTGAGAACGGGAA-3’
	R:5’-AAATGAGCCCCAGCCTTCTC-3’

### Network pharmacology analysis

To systematically elucidate the potential mechanisms underlying the therapeutic effects of YPTQ on AR, a network pharmacology analysis was conducted following established guidelines for rigorous evaluation. This approach integrates data from multiple sources to construct and analyze biological networks linking drug components, targets, and diseases. First, disease-related genes associated with AR were retrieved from three public databases: the Genecards (https://www.genecards.org/), the Comparative Toxicogenomics Database (CTD) (https://ctdbase.org/), and DisGeNET (https://www.disgenet.org/) databases. The retrieved gene lists were integrated and deduplicated to establish a comprehensive AR-related gene set. The active chemical constituents of each herbal medicine contained in the YPTQ formula were identified from the Traditional Chinese Medicine Systems Pharmacology Database and Analysis Platform (TCMSP, https://tcmsp-e.com/) and PubChem (https://pubchem.ncbi.nlm.nih.gov/). The common targets between YPTQ and AR were identified via intersecting the drug target set with the AR-related gene set using a Venn diagram. Gene Ontology (GO) functional annotation and Kyoto Encyclopedia of Genes and Genomes (KEGG) pathway enrichment analyses were performed on the common targets using the clusterProfiler R package (version 4.8.3). Terms with an adjusted *p*-value <0.05 were considered statistically significant. A protein-protein interaction (PPI) network for the common targets was constructed using the STRING database (https://string-db.org/, version 12.0), with a minimum required interaction score (confidence) set to > 0.4. The resulting network was downloaded and imported into Cytoscape software (version 3.9.0) for visualization and topological analysis. To identify key targets, the following topological thresholds were applied: degree centrality ≥ 3, betweenness centrality > 0.00001, and closeness centrality > 0.37. GO functional enrichment analysis was then performed on these key targets, and the top 11 significantly enriched pathways (adjusted *p* < 0.05) were selected for subsequent analysis. To visualize the multi-level relationships, a “herb-target-pathway” network was built by integrating compound-target mappings from DrugBank, TTD, ChEMBL, and PubChem databases, and target gene names were standardized using UniProt. The resulting multi-layered interaction network was constructed and visualized using Cytoscape.

### Molecular docking

Molecular docking was performed using AutoDock Vina 1.2.0 (24) to evaluate the binding affinities between the key active ingredients of YPTQ and critical targets, including TSLP and AhR. The crystal structures of TSLP (PDB ID: 5J13) and AHR (PDB ID: 8QMO) were obtained from the Protein Data Bank (https://www.rcsb.org/) (25). The protein structures were prepared by removing water molecules and co-crystallized ligands, adding hydrogen atoms, and assigning Gasteiger charges using AutoDockTools (version 1.5.7). The prepared structures were saved in PDBQT format. The 3D chemical structures of the small molecules were downloaded from PubChem database (https://pubchem.ncbi.nlm.nih.gov/) (26) in SDF format. These structures were converted to MOL2 format using Open Babel 2.4.1, followed by energy minimization with PyRx 0.8. Docking simulations were carried out using AutoDock Vina (version 1.2.0). A binding energy ≤−5 kcal/mol was considered indicative of a strong and potentially significant interaction (27). Finally, the molecular interaction patterns between the compound and the receptor proteins were visualized using PyMOL (28).

### Ahr luciferase reporter assay

The transcriptional activity of AhR was assessed using a luciferase reporter gene assay in A549 cells (a human alveolar epithelial cell line). A549 cells were cultured in 96-well black-rimmed, transparent-bottom plates at a density of 2 × 10^5^ cells/mL. Cells were transfected with an AhR-responsive luciferase plasmid (Servicebio, Wuhan, China) using Lipofectamine 3000 Transfection Kit (Thermo Fisher Scientific, USA), following the manufacturer's protocol. After 24 h of transfection, the cells were treated with YPTQ or kaempferol at specified concentrations for an additional 24 h. Luciferase activity was measured using the ONE-Glo^TM^ Luciferase Assay System (Promega Corporation, USA) on a multimode microplate reader (BioTek, USA).

### siRNA transfection

To knock down the expression of specific target genes, small interfering RNAs (siRNAs) were employed. siRNAs targeting human TSLP (siTSLP) and AhR (siAhR) mRNAs, along with a non-targeting negative control siRNA (NC), were designed and synthesized by GenScript (Jiangsu, China). RPMI-2650 cells were transfected with 25 nM of the respective siRNA using Lipofectamine™ RNAiMAX (Thermo Fisher Scientific, USA) according to the manufacturer's instructions. Cells were incubated for 24 h post-transfection before subsequent treatments or analyses. Knockdown efficiency was verified by RT-qPCR.

### Dendritic cell (DC) migration assay

A transwell migration assay was used to evaluate the chemotactic effect of conditioned medium from treated nasal epithelial cells on dendritic cells. Mouse bone marrow derived dendritic cells (BMDCs) were isolated as previously described (29) and seeded in the upper chamber of a transwell plate system. RPMI-2650 cells were treated with YPTQ extract or vehicle control. The conditioned medium was then collected and placed in the lower chamber of the transwell plate. After incubation at 37 °C for 6 h, cells that had migrated through the membrane to the lower surface were fixed with 4% paraformaldehyde for 30 min and stained with 0.1% crystal violet for 20 min. Non-migrated cells on the upper surface of the membrane were carefully removed with a cotton swab. The stained migrated cells on the lower membrane surface were imaged under an inverted light microscope. Cells from five randomly selected fields per well were counted, and the average number was calculated.

### Statistical analysis

All quantitative data are presented as the mean ± standard deviation (SD). Statistical analyses were performed using GraphPad Prism (GraphPad Software, USA). comparisons between two groups were determined using *t*-tests, while comparisons among multiple groups were determined using one-way analysis of variance (ANOVA) followed by Tukey's *post hoc* test. *P* < 0.05 was considered statistically significant.

## Results

### YPTQ ameliorates OVA-induced AR pathology and inflammatory cell infiltration in AR rats

**Figure 1:**
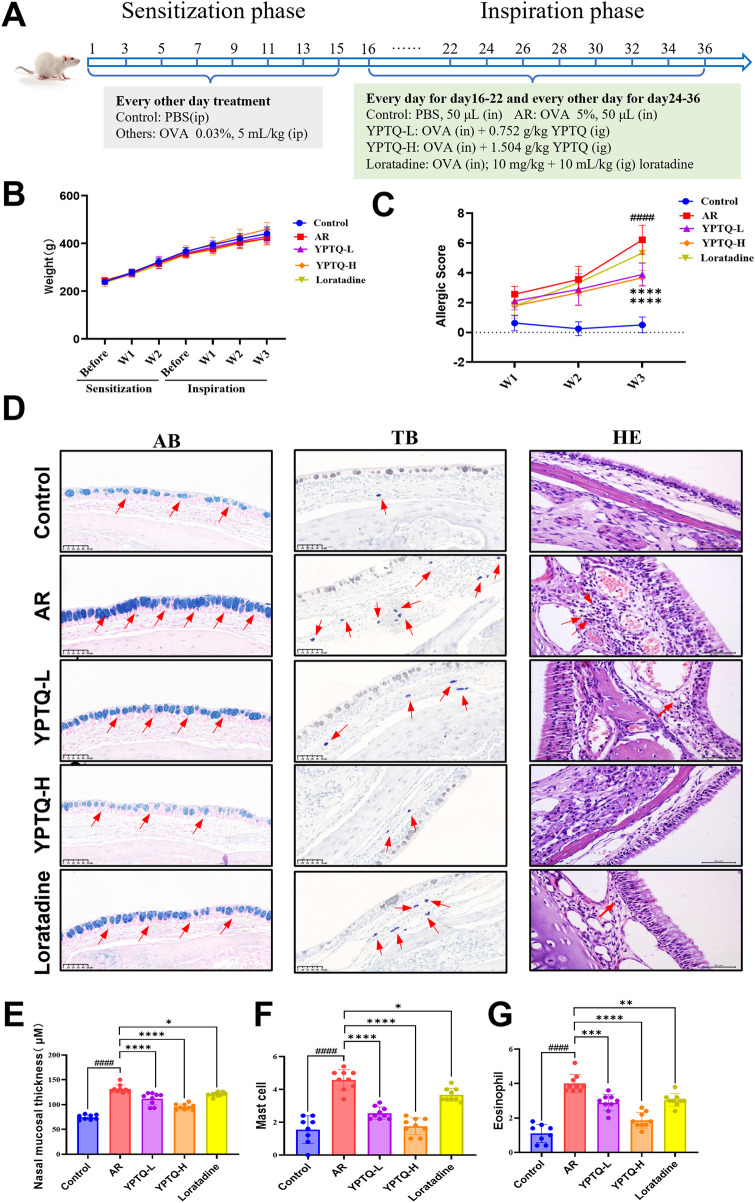
YPTQ mitigated OVA-induced nasal pathological injury in AR rats. **(A)** Schematic diagram illustrating the experimental design for establishing the AR model and drug administration regimen. **(B)** Body weight changes across different experimental groups during experimental stage. **(C)** Assessment of nasal allergy symptom scores. **(D)** Representative images of nasal tissue stained with AB staining (left panel, red arrows point mucosal layer), TB staining (middle panel, red arrows point mast cells) and H&E staining (right panel, red arrows point eosinophils). Scale bar: 50 μm. **(E,F)** Quantitative analysis of nasal mucosal thickness (E, derived from AB staining of panel D), mast cells (F, derived from TB staining of panel D) and eosinophils (G, derived from H&E staining of panel D). Data are presented as mean ± SD. ^####^*P* < 0.0001 vs. the control group; *P < 0.05, **P < 0.01, ***P < 0.001, ****P < 0.0001 vs. the AR group.

To evaluate the therapeutic effect of YPTQ, an OVA-induced AR rat model was established following a well-established two-phase sensitization and inspiration protocol (21, 22). The model was induced by intraperitoneal (i.p.) sensitization with OVA from days 1–15, followed by local intranasal (i.n.) OVA challenge from days 16–36 (Figure 1A). YPTQ was administered intragastrically (i.g.) starting from the first day of the challenge phase. Throughout the experimental period, body weight was monitored, and no significant differences were observed among all groups (Figure 1B), indicating that neither the AR modeling procedure nor the drug interventions adversely affected the general health of the animals. Nasal allergy scores were significantly higher in the AR model group than in the normal control group during the challenge phase (Figure 1C), confirming successful model induction. Of note, treatment with YPTQ, but not with the positive control drug loratadine, significantly reduced these allergy scores compared with the AR model group (Figure 1C), indicating a superior therapeutic effect of YPTQ. Moreover, histopathological (AB, TB, H&E) staining of nasal mucosal tissues revealed characteristic AR-related pathological changes in the model group (Figure 1D), including significant nasal mucosal thickening (Figure 1E). Specific staining for inflammatory cells showed a marked increase in the infiltration of mast cells (Figure 1F) and eosinophils (Figure 1G) in the AR group compared to the control group. In contrast, both YPTQ and loratadine treatments significantly attenuated these pathological alterations, as evidenced by reduced mucosal thickness, and decreased numbers of infiltrated mast cells and eosinophils (Figures 1D–G). Importantly, YPTQ exerted superior efficacy compare to loratadine (Figures 1D–G).

### YPTQ suppresses OVA-induced inflammatory responses in AR rats

**Figure 2:**
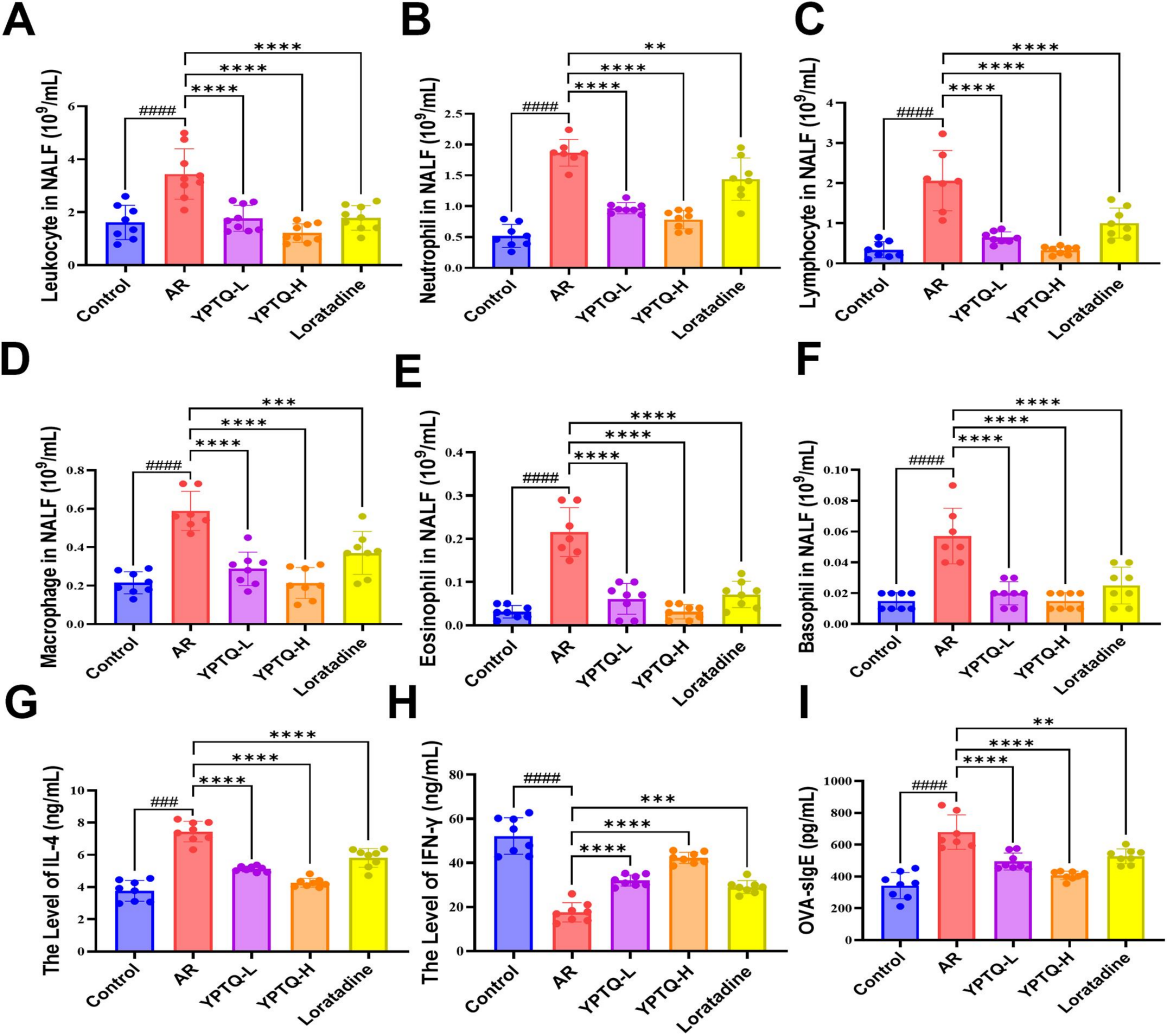
YPTQ mitigated OVA-induced inflammatory response in AR rats. (A–F) Differential counts of inflammatory cells, including Leukocytes **(A)**, neutrophils **(B)**, lymphocytes **(C)**, macrophages **(D)**, eosinophils **(E)**, and basophils **(F)** in NALF. (**G–I**) Serum levels of OVA-sIgE **(G)**, IL-4 **(H)** and IFN-*γ* **(I)** quantified by ELISA. Data are presented as mean ± SD. ^###^*P* < 0.001, ^####^*P* < 0.0001 vs. the control group; **P < 0.01, ***P < 0.001, ****P < 0.0001 vs. the AR group.

Having established that YPTQ alleviates the pathological features of AR, we next investigated its impact on the underlying inflammatory response, which is a core driver of AR pathogenesis (9). The leukocyte counts in NALF revealed a comprehensive inflammatory cell influx in AR. Compared to the control group, the AR group exhibited significant increases in the numbers of total leukocyte (Figure 2A), neutrophils (Figure 2B), lymphocytes (Figure 2C), macrophages (Figure 2D), eosinophils (Figure 2E), and basophils (Figure 2F). Treatment with both YPTQ and loratadine resulted in marked reductions in all these inflammatory cell populations compared to the AR model group (Figures 2A–F). Of note, YPTQ exhibited a superior efficacy compare to loratadine (Figures 2A–F).

Further analysis of cytokine profiles in NALF via ELISA showed that AR induction led to a significant upregulation of the Th2 cytokine IL-4 (Figure 2G) and a concomitant downregulation of the Th1 cytokine IFN-*γ* (Figure 2H), indicating a Th2-skewed immune response. Both YPTQ and loratadine treatments effectively reversed these cytokine imbalances (Figures 2G,H). Moreover, serum levels of OVA-specific immunoglobulin E (OVA-sIgE), a key mediator in type I hypersensitivity reactions, were significantly elevated in AR rats (Figure 2I). YPTQ and loratadine treatments effectively attenuated the alterations of cytokines and OVA-sIgE (Figures 2H,I). Collectively, these results demonstrate that YPTQ effectively suppresses OVA-induced inflammatory responses in AR rats.

### YPTQ attenuates HDM-induced inflammatory responses and protects nasal epithelial barrier integrity *in vitro*

**Figure 3:**
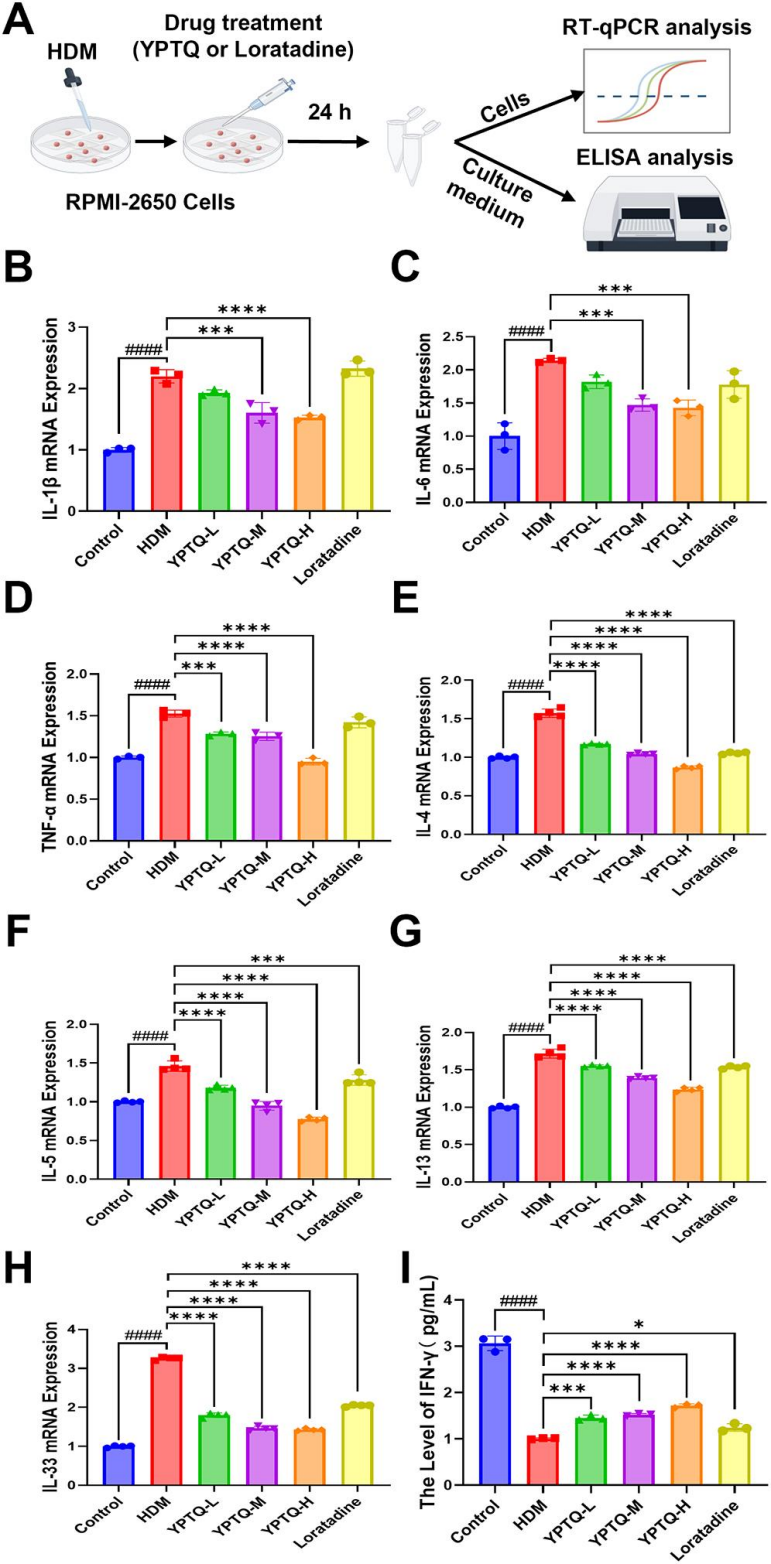
YPTQ attenuated HDM-induced inflammatory cytokines expression in RPMI-2650 cells. **(A)** Schematic representation of the experimental design for HDM stimulation and YPTQ treatment. **(B–H)** Relative mRNA levels of inflammatory cytokines, including IL-1β **(B)**, IL-6 **(C)**, TNF-α **(D)**, IL-4 **(E)**, IL-5 **(F)**, IL-13 **(G)** and IL-33 **(H)**, were measured by RT-qPCR in RPMI-2650 cells. **(I)** Protein levels of IFN-*γ* in the supernatant of RPMI-2650 cells quantified by ELISA. Data are presented as mean ± SD. ####*P* < 0.0001 vs. the control group; *P < 0.05, ***P < 0.001, ****P < 0.0001 vs. the HDM group.

To further elucidate the mechanism by which YPTQ mitigates inflammatory responses in nasal epithelial cells, an *in vitro* model of HDM-induced nasal epithelial injury was established using the human nasal epithelial cell line RPMI-2650 (Figure 3A). RT-qPCR analysis demonstrated that HDM stimulation significantly upregulated the mRNA expression of a broad spectrum of inflammatory cytokine levels, including pro-inflammatory cytokines (IL-1β, IL-6, TNF-α), Th2 cytokines (IL-4, IL-5, IL-13), and the alarmin IL-33 (Figures 3B–H). YPTQ treatment dose-dependently reversed HDM-induced upregulation of all these cytokines (Figures 3B–H). Notably, loratadine primarily inhibited the expression of Th2 cytokines and IL-33 but showed limited effect on the pro-inflammatory cytokines IL-1β, IL-6, and TNF-α (Figures 3B–H). In addition, ELISA analysis revealed that HDM exposure significantly decreased the secretion of IFN-*γ* (Figure 3I), and YPTQ treatment dose-dependently restored its levels, showing a more pronounced effect than loratadine (Figure 3I).

**Figure 4:**
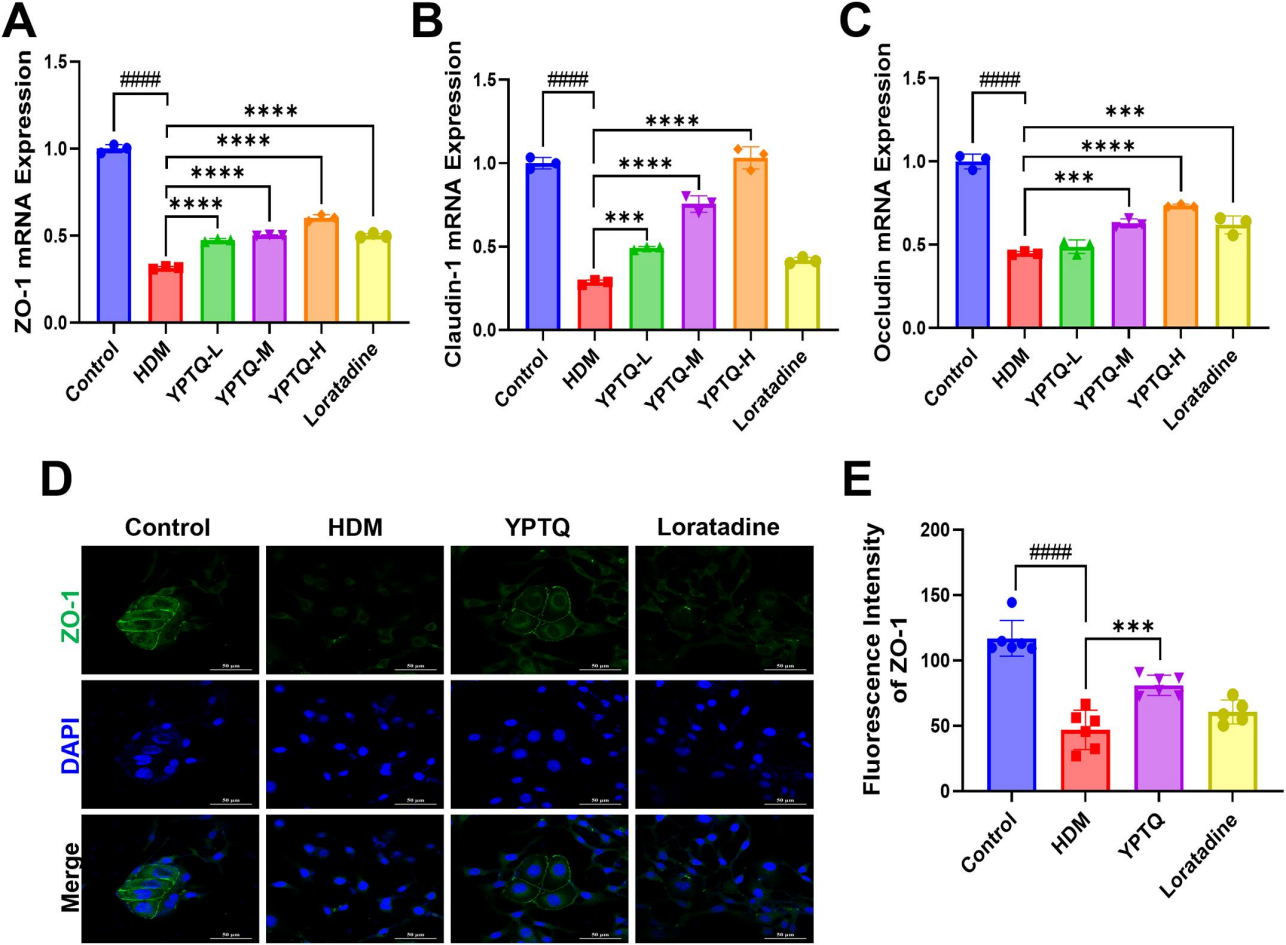
YPTQ ameliorated HDM-induced downregulation of tight junction protein expression in RPMI-2650 cells. (**A–C**) Relative mRNA levels of ZO-1, Claudin-1 and Occludin genes analyzed by RT-qPCR in RPMI-2650 cells. (**D–E**) Representative IF staining of ZO-1 (green) in RPMI-2650 cells (400×). Scale bar: 50 μm. Data are presented as mean ± SD. ^####^*P* < 0.0001 vs. the control group; ***P < 0.001, ****P < 0.0001 vs. the HDM group.

Given the critical role of the nasal epithelial barrier as the first line of defense, we next evaluated the protective effect of YPTQ on tight junction proteins, which are essential for maintaining barrier integrity. HDM stimulation significantly downregulated the transcriptional levels of tight junction proteins ZO-1 (Figure 4A), Claudin-1 (Figure 4B), and Occludin (Figure 4C). In contrast, YPTQ treatment dose-dependently restored the transcriptional levels of these proteins, with a more potent effect than loratadine (Figures 4A–C). Further IF staining for ZO-1 protein revealed that HDM exposure led to a marked reduction of ZO-1 protein levels, which was significantly reversed by YPTQ but not loratadine administration (Figures 4D,E).

Collectively, these results showed that YPTQ has positive protective effects not only in attenuating HDM-induced inflammatory responses but also in effectively protecting against allergen-induced nasal epithelial barrier dysfunction by upregulating the expression of critical tight junction proteins.

### Network pharmacological analysis reveals TSLP and AhR as potential targets of YPTQ in AR

**Figure 5:**
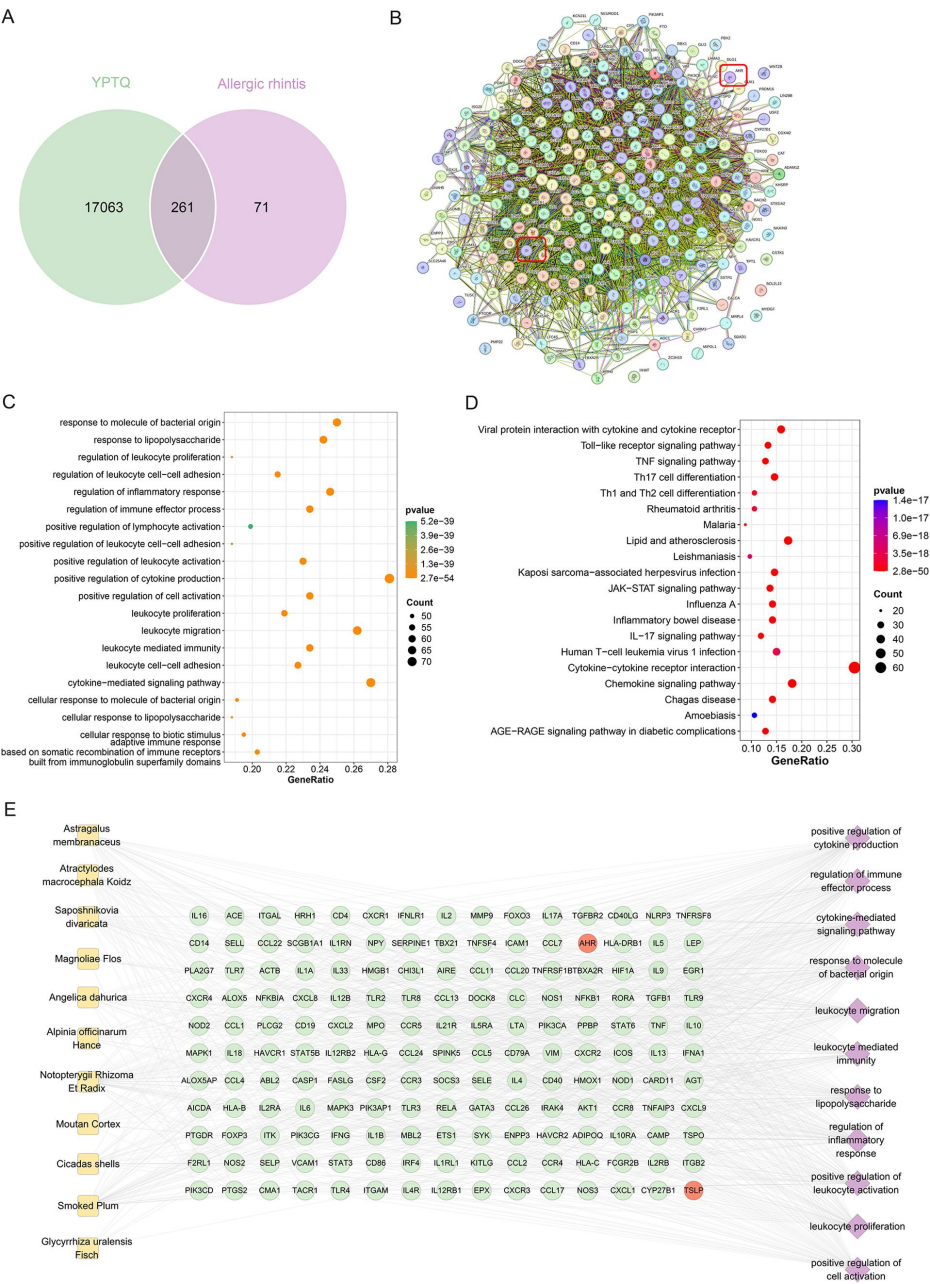
Network pharmacological analysis identified potential therapeutic targets and mechanisms of YPTQ against AR. **(A)** Venn diagram illustrating the overlapping targets between the active compounds of YPTQ and AR-related genes. **(B)** PPI network constructed from the intersecting targets identified in **(A)**, with node size and color intensity reflecting the degree of connectivity. (**C,D**) Bubble plot displaying the top 20 enriched GO terms **(C)** and significant enriched KEGG pathways **(D) (E)** A comprehensive “herb-target-pathway” association network. Nodes represent YPTQ herbs (yellow squares), key targets (green and red circles), and enriched GO pathways (purple diamonds). Key targets were selected based on topological analysis with thresholds: degree ≥ 3, betweenness > 0.00001, and closeness > 0.37. Only the top 11 enriched pathways (adjusted *p* < 0.05) are shown. Node size reflects degree centrality, and edge thickness represents interaction confidence.

To systematically elucidate the multi-component, multi-target therapeutic basis of YPTQ in ameliorating inflammatory responses and epithelial barrier dysfunction in AR, a comprehensive network pharmacology analysis was conducted (Figure 5). The intersection analysis between the potential targets of YPTQ and the genes associated with AR yielded 261 common genes (Figure 5A, Table 2), suggesting a broad regulatory scope of YPTQ on AR-related pathological processes. Subsequent protein-protein interaction (PPI) network analysis of these common targets revealed a highly interconnected network. Several genes, including TSLP, AHR, TNF, IL10, IL1B, CD4, CCL2, IFNG, STAT3, and CXCL8, exhibited high degree centrality, indicating their pivotal roles as potential hub genes within the network (Figure 5B). To functionally characterize these common targets, Gene Ontology (GO) and Kyoto Encyclopedia of Genes and Genomes (KEGG) pathway enrichment analyses were performed. The GO enrichment analysis indicated that these targets were primarily involved in key biological processes (BP) related to immune dysregulation in AR, such as leukocyte-mediated immunity, positive regulation of cytokine production, and regulation of inflammatory response (Figure 5C). Consistently, KEGG pathway analysis demonstrated that these genes were significantly enriched in pathways crucial for immune and inflammatory regulation, including cytokine-cytokine receptor interaction, chemokine signaling pathway, Th17 cell differentiation, and TNF signaling pathway (Figure 5D).

**Table 2:** Intersection targets of YPTQ and AR.

No	Target	No	Target	No	Target	No	Target	No	Target	No	Target
1	ABCA1	45	CD19	89	GAS5	133	IL31	177	NKAIN3	221	SOCS3
2	ABL2	46	CD3D	90	GATA3	134	IL33	178	NLRP3	222	SPINK5
3	ACE	47	CD4	91	GLI3	135	IL4	179	NOD1	223	SPP1
4	ACTB	48	CD40	92	GSDMB	136	IL4R	180	NOD2	224	SSTR1
5	ADAM12	49	CD40LG	93	GSTK1	137	IL5	181	NOS1	225	ST8SIA2
6	ADAM33	50	CD79A	94	HAVCR1	138	IL5RA	182	NOS2	226	STAT3
7	ADIPOQ	51	CD86	95	HAVCR2	139	IL6	183	NOS3	227	STAT5B
8	ADRB2	52	CFD	96	HCLS1	140	IL9	184	NPY	228	STAT6
9	AGT	53	CHI3L1	97	HCP5	141	IRAK4	185	NR3C1	229	SYK
10	AHR	54	CHRM3	98	HDC	142	IRF4	186	ORMDL3	230	TAC1
11	AICDA	55	CLC	99	HHAT	143	ISG20	187	PBX2	231	TACR1
12	AIRE	56	CMA1	100	HIF1A	144	ITGAL	188	PDE4A	232	TAP1
13	AKT1	57	COX4I2	101	HLA-B	145	ITGAM	189	PGM3	233	TAP2
14	ALB	58	CSF2	102	HLA-C	146	ITGB2	190	PHF11	234	TBX21
15	ALOX5	59	CUX1	103	HLA-DRB1	147	ITK	191	PIK3AP1	235	TBXA2R
16	ALOX5AP	60	CXCL1	104	HLA-G	148	JUN	192	PIK3CA	236	TGFB1
17	AOC1	61	CXCL2	105	HMGB1	149	KCNJ11	193	PIK3CB	237	TGFBR2
18	BACH2	62	CXCL8	106	HMOX1	150	KHSRP	194	PIK3CD	238	TIMP1
19	BCL2L12	63	CXCL9	107	HNMT	151	KITLG	195	PIK3CG	239	TLR2
20	BDNF	64	CXCR1	108	HRH1	152	KNG1	196	PIP	240	TLR3
21	BLK	65	CXCR2	109	HRH3	153	LAMA3	197	PLA2G7	241	TLR4
22	CALCA	66	CXCR3	110	HRH4	154	LEP	198	PLAT	242	TLR7
23	CAMP	67	CXCR4	111	ICAM1	155	LTA	199	PLCG2	243	TLR8
24	CARD11	68	CYSLTR1	112	ICOS	156	LTC4S	200	PMP22	244	TLR9
25	CASP1	69	DEFB1	113	IFNA1	157	MAPK1	201	PPBP	245	TNF
26	CAT	70	DLG1	114	IFNG	158	MAPK3	202	PRDM16	246	TNFAIP3
27	CCL1	71	DNAH5	115	IFNLR1	159	MBL2	203	PSIP1	247	TNFRSF1B
28	CCL11	72	DOCK8	116	IL10	160	MIPOL1	204	PTGDR	248	TNFRSF8
29	CCL13	73	EGR1	117	IL10RA	161	MIR126	205	PTGDR2	249	TNFSF4
30	CCL17	74	EMSY	118	IL12B	162	MIR143	206	PTGS2	250	TPT1
31	CCL2	75	ENPP3	119	IL12RB1	163	MIR146A	207	RBX1	251	TRPV1
32	CCL20	76	EPS15	120	IL12RB2	164	MIR155	208	RELA	252	TSLP
33	CCL22	77	EPX	121	IL13	165	MIR21	209	RNASE3	253	TSPO
34	CCL24	78	ETS1	122	IL16	166	MMP9	210	RORA	254	TUSC1
35	CCL26	79	F2RL1	123	IL17A	167	MPO	211	RORC	255	USF2
36	CCL27	80	FASLG	124	IL18	168	MRPL4	212	SCGB1A1	256	VCAM1
37	CCL4	81	FCGR2B	125	IL1A	169	MUC5AC	213	SDAD1	257	VDR
38	CCL5	82	FLG	126	IL1B	170	MYDGF	214	SELE	258	VEGFA
39	CCL7	83	FLNC	127	IL1RL1	171	NEAT1	215	SELL	259	VIM
40	CCR3	84	FN1	128	IL1RN	172	NEUROD1	216	SELP	260	VIP
41	CCR4	85	FOXJ1	129	IL2	173	NFE2L2	217	SERPINE1	261	WNT2B
42	CCR5	86	FOXO3	130	IL21R	174	NFKB1	218	SLC25A46		
43	CCR8	87	FOXP3	131	IL2RA	175	NFKBIA	219	SLC3A2		
44	CD14	88	FTO	132	IL2RB	176	NGF	220	SNHG16		

To further clarify the specific regulatory relationships, a core “herb-target-pathway” interaction network was established based on association rules and topological analysis (Figure 5E). This integrative network provides a systemic perspective on how the active ingredients of YPTQ may collectively modulate AR pathology through interconnected targets and pathways. Among these identified hub genes, AhR and TSLP were prioritized for further investigation due to their established critical roles: AhR is known to disrupt epithelial barrier integrity, whereas TSLP drives type 2 inflammation (11).

**Figure 6:**
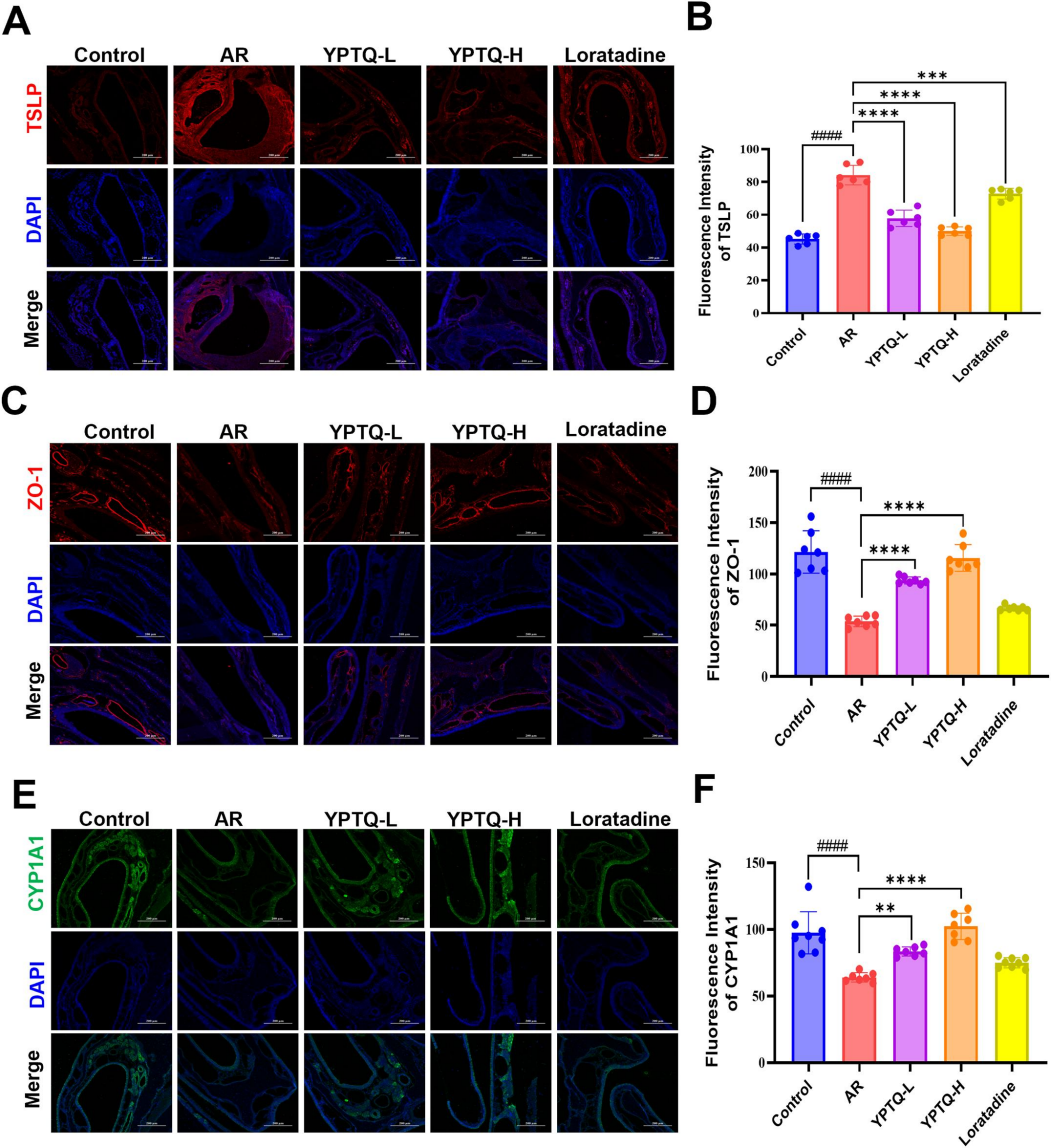
YPTQ attenuated OVA-triggered upregulation of TSLP and downregulation of ZO-1 and CYP1A1 in AR rats. (**A–F**) Representative IF staining images **(A,C,E)** and corresponding quantitative analysis **(B,D,F)** of TSLP (red) (A and B), ZO-1 (red) (C and D), CYP1A1 (green) (E and F) proteins in nasal tissues (100×). Scale bar: 200 μm. Data are presented as mean ± SD. ^####^*P* < 0.0001 vs. the control group; **P < 0.01, ***P < 0.001, ****P < 0.0001 vs. the AR group.

Based on the network pharmacology predictions, we hypothesized that YPTQ exerts its therapeutic effects by simultaneously inhibiting the upstream inflammatory modulator TSLP and activating the AhR signaling pathway, which is known to reinforce epithelial barrier function. To test this, we subsequently evaluated the protein expression of TSLP, the tight junction protein ZO-1 (a marker of epithelial integrity), and CYP1A1 [a canonical downstream target gene of AhR (30)] in the nasal mucosal tissues of rats from different treatment groups using IF staining. Consistent with the AR pathology, IF staining revealed a significant upregulation of TSLP expression in the nasal mucosa of the AR model group compared to the control group (Figures 6A,B). Treatment with YPTQ markedly reduced this elevation, with superior inhibitory effect compare to that observed in the loratadine-treated group (Figures 6A,B). Conversely, the expression levels of ZO-1 (Figures 6C,D) and CYP1A1 (Figures 6E,F) were significantly decreased in the AR group. Notably, administration of YPTQ effectively restored the expression of both ZO-1 and CYP1A1, whereas loratadine treatment showed no significant effect on these proteins (Figures 6C–F). Together, these results validate the network pharmacology predictions and demonstrate that TSLP and AhR are potential targets of YPTQ in regulating immune responses and maintaining nasal epithelial barrier integrity in AR. Therefore, we focused on validating and deciphering the underlying mechanisms of YPTQ via TSLP and AhR/CYP1A1 signaling pathways.

### YPTQ ameliorates AR inflammatory responses and DC cell migration via suppressing TSLP signaling *in vitro*

**Figure 7:**
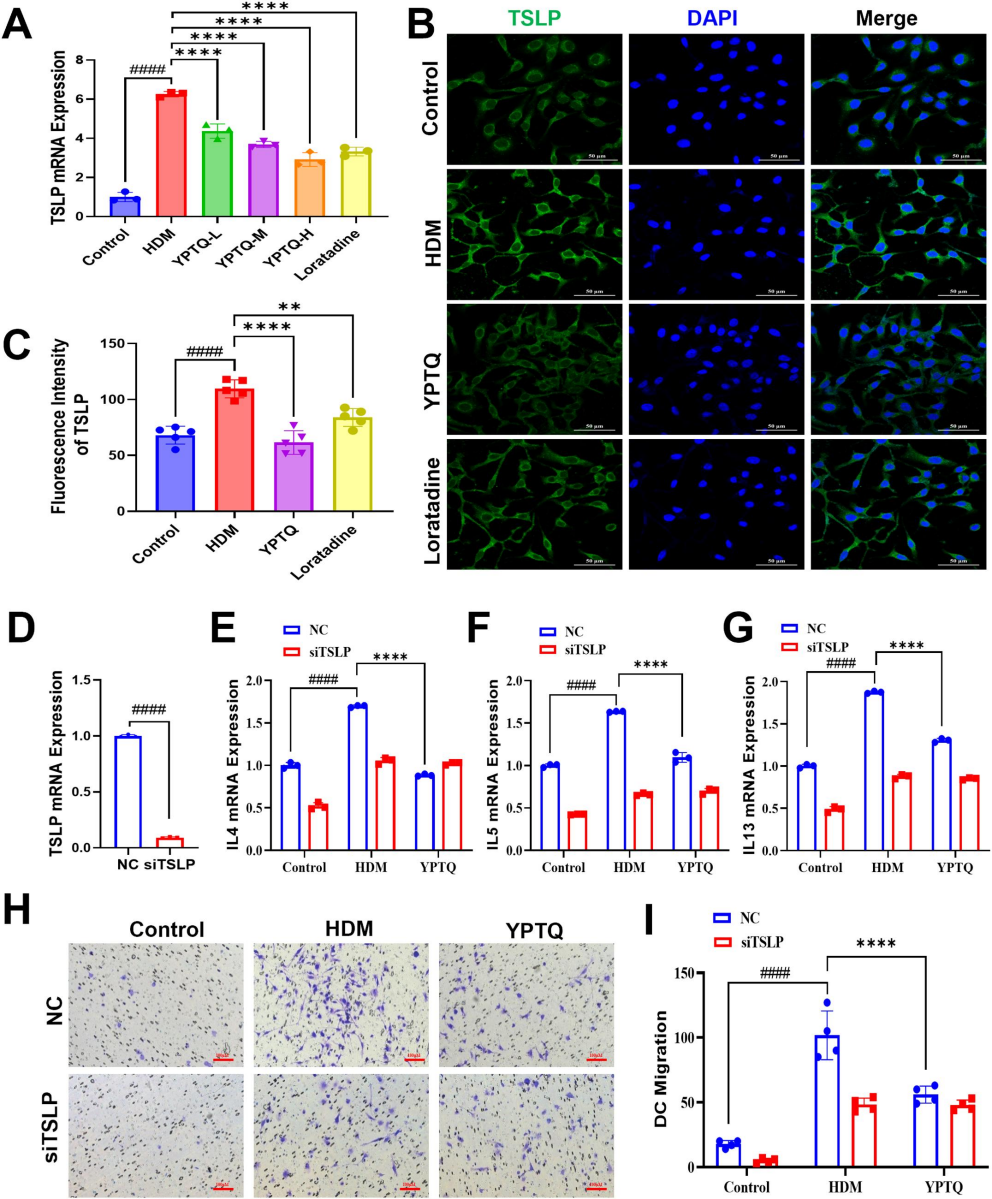
YPTQ ameliorated inflammatory response by modulating the TSLP signaling pathway in HDM induced RPMI-2650 cell injury model. **(A)** Relative mRNA levels of TSLP genes in RPMI-2650 cells measured by RT-qPCR. **(B,C)** Representative IF staining images of TSLP (green) in RPMI-2650 cells (400×) Scale bar: 50 μm. **(D)** Relative mRNA expression levels of TSLP in RPMI-2650 cells transfected with negative control siRNA (NC) or siRNA targeting TSLP (siTSLP). **(E–G)** Relative mRNA expression levels of IL-4 **(E)**, IL-5 **(F)** and IL-13 **(G)** in RPMI-2650 cells of control, HDM or YPTQ group, transfected with NC or siTSLP. **(H,I)** Evaluation of DC migration using a Transwell assay. Conditioned medium was collected from RPMI-2650 cells of control, HDM and YPTQ group, which has been transfected with NC or siTSLP. Representative images **(H)** and quantitative results **(I)** of migrated DCs stained with crystal violet. Scale bar: 100 μm. Data are presented as mean ± SD. ^####^*P* < 0.0001 vs. the control group; **P < 0.01, ****P < 0.0001 vs. the HDM group.

Next, to validate the transcription and expression levels of TSLP in an HDM-triggered nasal epithelial injury model in RPMI-2650 cells, RT-qPCR and IF staining were performed across different treatment groups (Figures 7A–C). Compared to the control group, both TSLP mRNA and protein levels were significantly upregulated in the HDM model group (Figures 7A–C). In contrast, treatment with either YPTQ or loratadine markedly attenuated this HDM-induced upregulation of TSLP (Figures 7A–C). Furthermore, to investigate whether the anti-inflammatory effects of YPTQ were mediated through TSLP, we employed siRNA to knock down TSLP expression in RPMI-2650 cells (Figure 7D). Subsequent RT-qPCR analysis revealed that TSLP knockdown abolished the suppressive effects of YPTQ on HDM-induced expression of the classic Th2 cytokines, including IL-4 (Figure 7E), IL-5 (Figure 7F), and IL-13 (Figure 7G).

Given that DC migration is a critical process in the initiation and amplification of allergic inflammation (31), we next evaluated whether YPTQ could inhibit HDM-triggered DC migration and whether this effect was dependent on TSLP signaling. To this end, DC migration assay was performed using conditioned medium collected from RPMI-2650 human nasal epithelial cells subjected to different treatments, including Control, HDM, and YPTQ groups, with or without TSLP knockdown. The results demonstrated that conditioned medium from HDM-stimulated RPMI-2650 cells significantly promoted DC migration, whereas YPTQ treatment significantly attenuated HDM-triggered DC migration in normal nasal epithelial cells without TSLP knockdown (NC group, Figures 7H,1). Intriguingly, the inhibitory effect of YPTQ on DC migration was completely abolished in cells where TSLP expression was knocked down (siTSLP groups, Figures 7H,1).

Collectively, these findings from both gene expression and functional assays demonstrate that the anti-inflammatory effects of YPTQ, including the suppression of Th2 cytokine production and the inhibition of DC migration, are mediated, at least in part, through the inhibition of the TSLP signaling pathway in nasal epithelial cells.

### YPTQ ameliorates nasal epithelial barrier dysfunction via activating AhR/CYP1A1 signaling pathway *in vitro*

**Figure 8:**
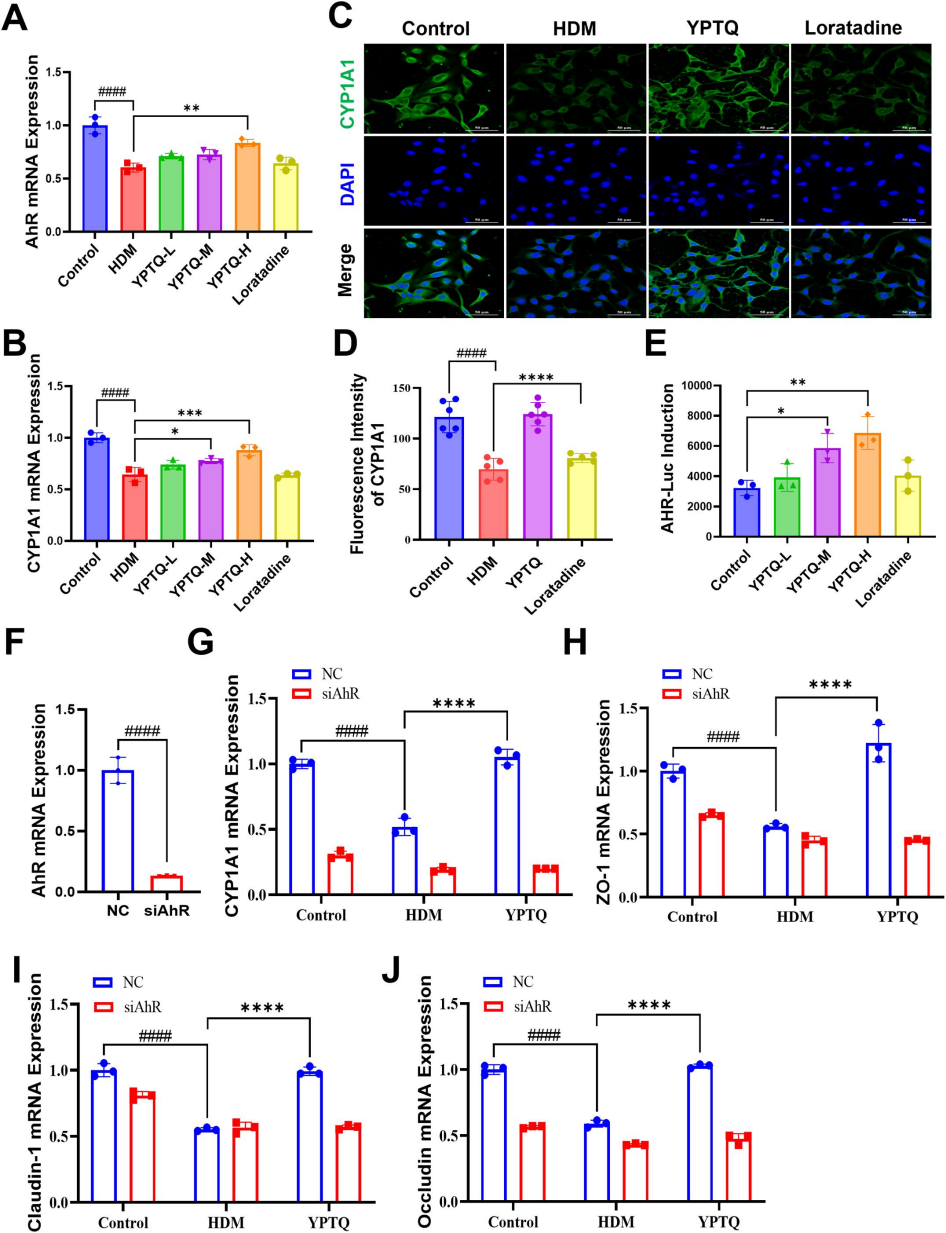
YPTQ preserved epithelial mucosal barrier through AhR/CYP1A1 signaling pathways in in HDM induced RPMI-2650 cell injury model. **(A,B)** Relative mRNA levels of AhR and CYP1A1 in RPMI-2650 cells measured by RT-qPCR. **(C,D)** Representative IF staining images **(C)** and quantitative results **(D)** of CYP1A1 (green) in RPMI-2650 cells (400×). Scale bar: 50 μm. **(E)** Activation efficiency of YPTQ on AhR signaling in A549 cells by AhR luciferase reporter assay. **(F)** Relative mRNA levels of AhR transfected with negative control siRNA (NC) or siRNA targeting AhR (siAhR). **(G–J)** Relative mRNA expression levels of CYP1A1 **(G)**, ZO-1 **(H)**, Claudin-1 **(I)** and Occludin **(J)** in RPMI-2650 cells of control, HDM and YPTQ group, transfected with NC or siAhR. Data are presented as mean ± SD. ^#^*P* < 0.05, ^##^*P* < 0.01, ^####^*P* < 0.0001 vs. the control group; *P < 0.05, **P < 0.01, ***P < 0.001, ****P < 0.0001 vs. the HDM group.

To further validate the role of YPTQ in regulating the AhR/CYP1A1 signaling pathway *in vitro*, we assessed the transcription levels of AhR and CYP1A1 by RT-qPCR and the protein expression levels of CYP1A1 by IF staining in an HDM-triggered nasal epithelial injury model in RPMI-2650 cells (Figures 8A–D). Compared to the control group, the HDM group exhibited significantly decreased mRNA levels of AhR and CYP1A1 (Figures 8A,B), as well as reduced protein levels of CYP1A1 (Figures 8C,D). Conversely, YPTQ treatment significantly reversed HDM-induced downregulation of both AhR and CYP1A1 at the transcriptional and protein levels (Figures 8A–D).

To provide direct evidence that YPTQ activates the AhR signaling pathway, we utilized an AhR luciferase reporter gene assay. The results showed that YPTQ activated AhR signaling pathway in a dose-dependent manner, whereas loratadine showed no such effect (Figure 8E). Additionally, siRNA-mediated knockdown of AhR (Figure 8F) in RPMI-2650 cells led to significant downregulation of CYP1A1 (Figure 8G) and tight junction proteins (ZO-1, Claudin-1, and Occludin) (Figures 8H–J), which effectively mimicked the HDM-induced barrier dysfunction and abrogated the therapeutic effects of YPTQ (Figures 8H–J). These findings collectively indicate that YPTQ improves nasal epithelial barrier function by activating the AhR/CYP1A1 signaling pathway.

### Kaempferol serves as a key active compound of YPTQ for its therapeutic effects via dual modulation of TSLP and AhR signaling in AR cellular model

**Figure 9:**
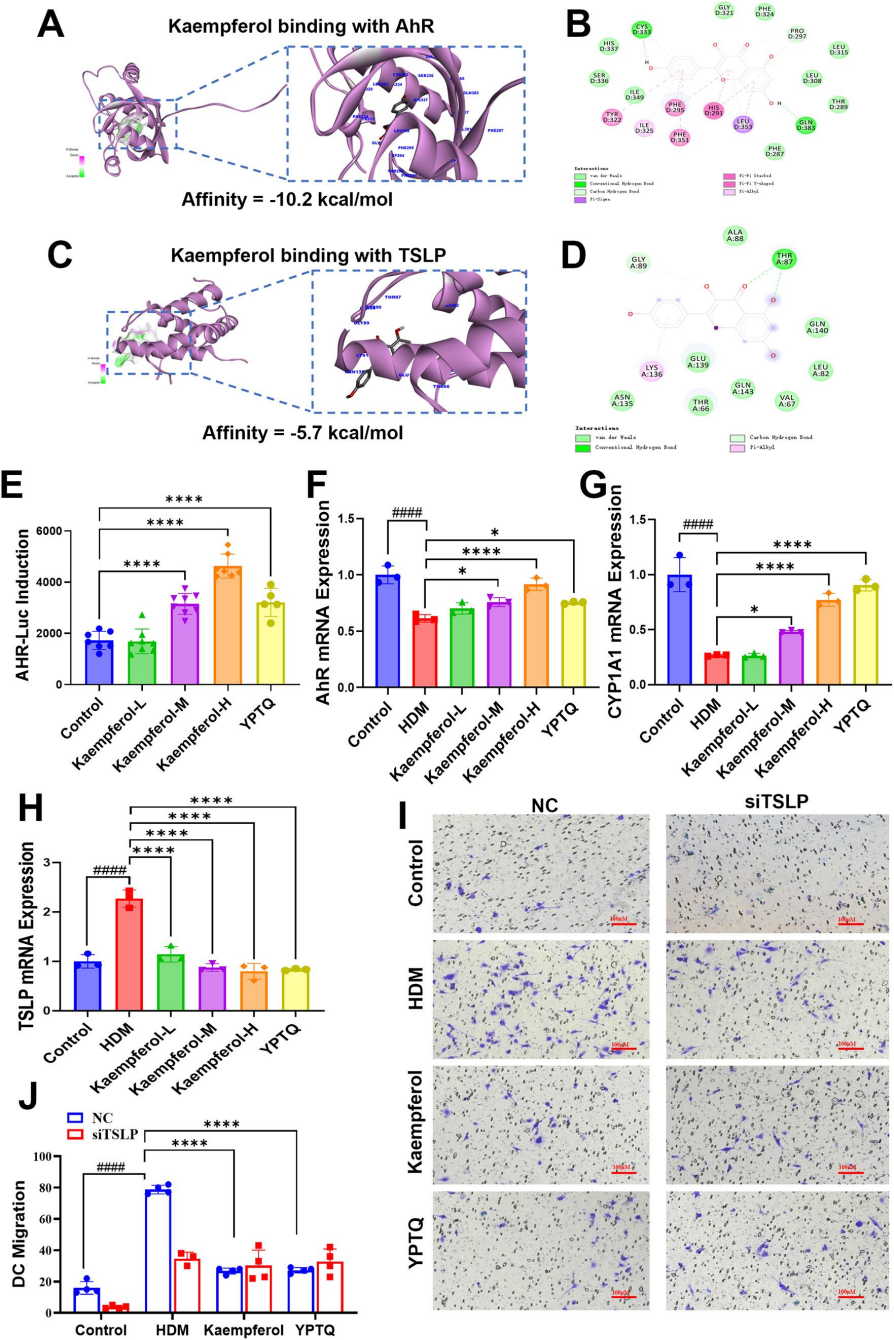
Molecular docking analysis and validation of therapeutic effects of kaempferol in RPMI-2650 cells induced by HDM. (**A–D**) Molecular docking analysis reveals the predicted binding mode of Kaempferol to **(A,B)** TSLP and AhR **(C,D)**. **(E)** AhR luciferase reporter assay indicating dose-dependently activation efficiency of Kaempferol on AhR signaling in A549 cells. (**F–H**) Relative mRNA levels of AhR **(F)**, CYP1A1 **(G)** and TSLP **(H)** in RPMI-2650 cells of different treatment groups measured by RT-qPCR. (**I,J**) Evaluation of DC migration using a Transwell assay. Conditioned medium was collected from RPMI-2650 cells of control, HDM, kaempferol and YPTQ group, which has been transfected with NC or siTSLP. Evaluation of DC migration using a Transwell assay. Representative images **(I)** and quantitative results **(J)** of migrated DCs stained with crystal violet. Scale bar: 100 μm **(D)** Quantitative analysis of the number of migrated DCs **(E)** Data are presented as mean ± SD. ^####^*P* < 0.0001 vs. the control group; *P < 0.05, ****P < 0.0001 vs. the HDM group.

Next, to identify the potential active compounds of YPTQ responsible for interacting with the predicted core targets, molecular docking was performed against the TSLP and AhR proteins. Strikingly, among the multiple compounds screened, kaempferol exhibited favorable binding affinity (≤−5.0 kcal/mol) with both AhR (Figures 9A,B) and TSLP (Figures 9C,D), suggesting that kaempferol is a pivotal active compound in YPTQ capable of directly engaging both key targets. Consistently, the AhR luciferase reporter assay confirmed that kaempferol dose-dependently activated the AhR signaling pathway (Figure 9E). Subsequent RT-qPCR analysis also indicated that kaempferol treatment dose dependently ameliorated HDM-triggered suppression of AhR and its downstream target CYP1A1 (Figures 9F,G), while suppressing the elevated expression of TSLP (Figure 9H). This dual regulatory capacity of kaempferol mirrors the core mechanism identified for the whole YPTQ formula. Furthermore, DC migration assay showed that kaempferol treatment significantly inhibited HDM-triggered DC migration (Figures 9I,J). Crucially, this inhibitory effect of kaempferol on DC migration was abolished when TSLP was knocked down in the epithelial cells using siRNA (Figures 9I,J), a result consistent with the observations in the YPTQ treatment group. In summary, these findings demonstrate that kaempferol is a key active compound of YPTQ responsible for its anti-inflammatory and barrier-protective effects by directly binding to and functionally modulating both the AhR and TSLP targets. This evidence positions kaempferol as a critical contributor to the multi-target, multi-pathway efficacy of YPTQ in treating AR.

## Discussion

**Figure 10:**
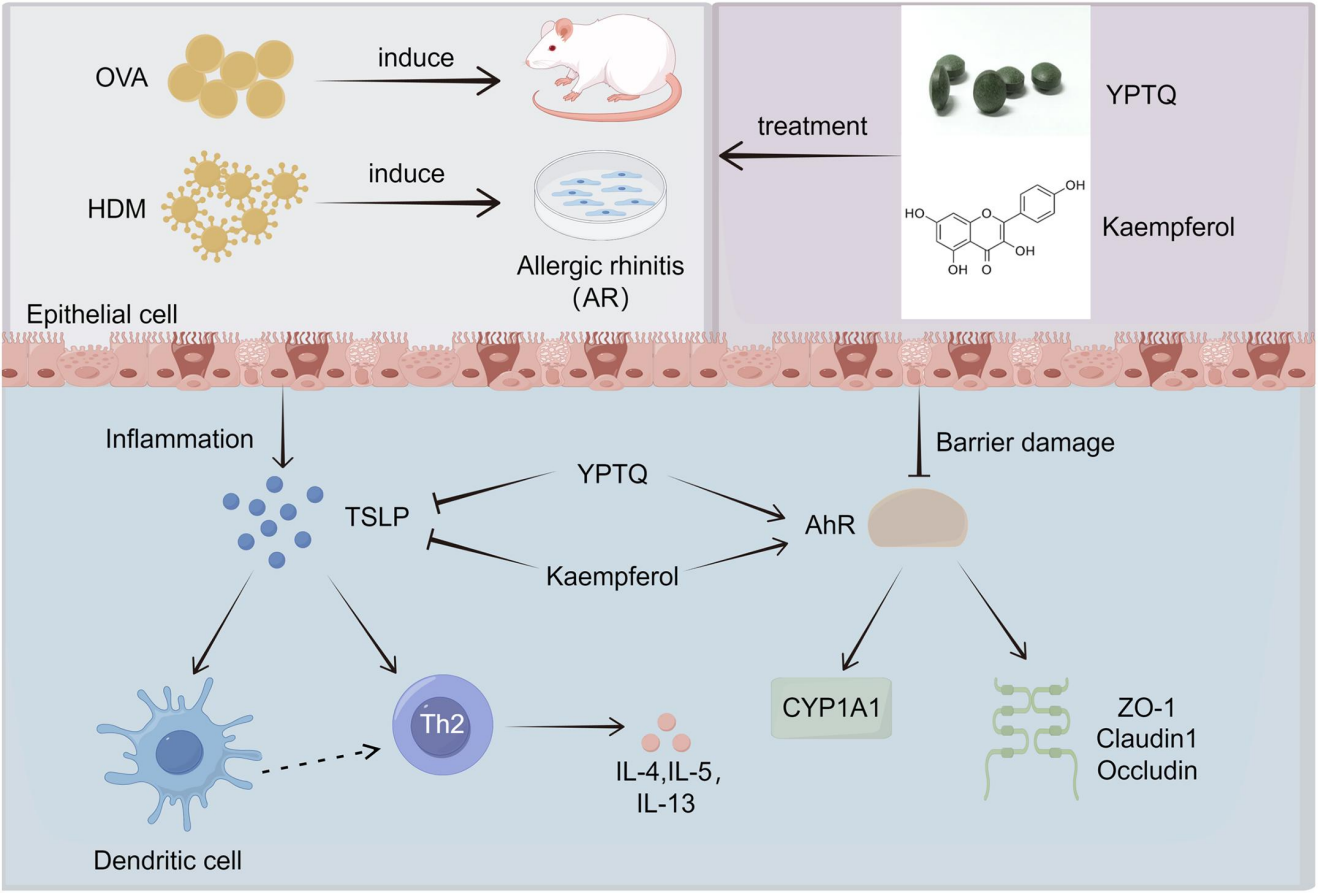
Graphical abstract. Multiple mechanisms of YPTQ against AR. YPTQ suppresses TSLP/DC-driven Th2 inflammation and activates AhR/CYP1A1-mediated epithelial barrier repair, with kaempferol serve as a key active compound. Created with Figdraw (https://www.figdraw.com).

AR is a chronic immunological disorder characterized by an inappropriate immune response to environmental allergens, leading to significant morbidity and impairment of quality of life (1, 2). This study demonstrates that YPTQ, a traditional Chinese medicine formula, exhibits excellent therapeutic effects on ameliorating OVA-induced AR pathology in rats by reducing the infiltration of various inflammatory cells (total leukocytes, neutrophils, lymphocytes, macrophages, eosinophils, basophils), correcting the Th2-skewed cytokine imbalance (reduced IL-4, increased IFN-*γ*), lowering serum OVA-specific IgE levels, and maintaining nasal epithelial barrier integrity via preserving tight junction protein (ZO-1, Claudin-1, and Occludin) expression. Mechanistically, we proved that YPTQ targets nasal epithelial cells to concurrently inhibit the pro-inflammatory TSLP pathway and activate the barrier-protective AhR/CYP1A1 signaling, thereby disrupting the epithelial-immune axis central to AR pathogenesis. The flavonoid kaempferol was identified as a key active component mediating this dual-target action. These findings not only elucidate a novel multi-target, multi-pathway pharmacological basis of YPTQ but also highlight the AhR and TSLP pathways as promising synergistic targets for AR therapy (Figure 10).

AR involves allergen-induced disruption of the nasal epithelial barrier, which triggers type 2 inflammatory responses and establishes a subsequent vicious cycle of “barrier damage-inflammation initiation” (10, 32). Current first-line pharmacological interventions for AR, such as oral antihistamines (e.g., loratadine) and leukotriene receptor antagonists (LTRAs) (33), primarily alleviate symptoms through blockade of specific mediators but do not directly restore epithelial barrier integrity, resulting in limited long-term efficacy (34). Consequently, interventions capable of targeting both inflammatory pathways and epithelial repair mechanisms are more likely to fundamentally mitigate core pathogenic processes of AR (32). In support of this concept, both prior evidence (20) and our current study, from clinical and non-clinical perspectives, demonstrate that YPTQ exerts significant anti-AR effects through multi-mechanistic actions. Importantly, our findings indicate that YPTQ exhibits superior efficacy to loratadine in correcting both core pathophysiological defects of AR, which is likely due to its multi-targeted mechanism. Loratadine, a conventional H₁-antihistamine agent, primarily blocks the downstream mediator histamine, thus providing symptomatic relief without addressing upstream inflammation processes or barrier repair. In contrast, YPTQ concurrently modulates inflammatory responses and enhances epithelial barrier homeostasis. Thus, compared with single-target agents such as loratadine, YPTQ represents a more comprehensive and mechanism-based therapeutic strategy for AR.

The core pathological feature of AR involves an imbalance in Th1/Th2 immune responses, characterized by a predominant Th2-type inflammatory reaction (35). This Th2 dominance leads to the overproduction of cytokines such as interleukin-4 (IL-4), IL-5, and IL-13, which drive the production of allergen-specific immunoglobulin E (IgE), eosinophil recruitment, and the classic symptoms of AR (36). Although this study did not directly measure the Th1/Th2 cytokine profile, our exploration of key upstream initiating events provides mechanistic clues for YPTQ's regulation of this imbalance. Specifically, YPTQ significantly suppressed the expression of TSLP, which is an epithelial-derived alarmin critical for initiating type 2 immunity. TSLP acts as a master switch, activating DCs and driving their migration to draining lymph nodes, which is a pivotal step in the activation and differentiation of naïve T cells into Th2 cells. Our study confirms that YPTQ effectively inhibits this DC migration, suggesting it may proximally block the initial sensitization and reactivation of allergen-specific Th2 cells, thereby mitigating the expansion of the Th2 immune response from its upstream origin. Concurrently, our study suggests YPTQ may activate the AhR pathway (37, 38), a vital regulator for maintaining epithelial barrier integrity and immune homeostasis, including the Th1/Th2 balance. By potentially enhancing barrier function through AhR activation, YPTQ could address a fundamental defect in AR, working synergistically with its suppression of the TSLP/DC axis. Collectively, we conclude that by targeting the core of the epithelial-immune axis (through suppressing TSLP and DC migration) and potentially synergistically enhancing barrier function (via AhR activation), YPTQ exerts broad-spectrum anti-inflammatory effects. This multi-pronged approach ultimately alleviates symptoms and pathological damage in the AR animal model. These findings provide novel and robust experimental evidence for a “multi-component, multi-target” intervention strategy, moving beyond symptomatic relief offered by current mainstay therapies such as antihistamines and corticosteroids, to correct the underlying Th1/Th2 imbalance in AR from multiple angles.

There are several limitations in this study that should be addressed in future research. First, the identification of active components in YPTQ for network pharmacology analysis was primarily based on database mining (TCMSP, PubChem) rather than analysis of *in vivo* blood-absorbed compounds using high-resolution mass spectrometry (HRMS). Although our subsequent molecular docking and functional assays confirmed the regulatory effects of YPTQ and kaempferol on TSLP and AhR, future studies precisely identify the *in vivo* absorbed components of YPTQ will further refine target prediction and enhance mechanistic understanding. Second, although kaempferol was identified as a key active compound in our study, we cannot conclude that it accounts for all the activity of YPTQ against multiple targets in AR (39, 40), given the “multi-component, multi-target” advantage philosophy of herbal medicine (41). Therefore, further investigation into the potential synergistic or additive effects among other active compounds within the YPTQ formula is warranted. Third, while AhR activation and TSLP inhibition were demonstrated, the precise upstream mechanism by which YPTQ or kaempferol activates AhR or suppresses TSLP (e.g., direct binding as a ligand, indirect modulation) was not fully elucidated. More detailed biochemical studies are needed. Additionally, while our preclinical models suggest that the dual regulatory capacity of kaempferol mirrors the core mechanism identified for the whole YPTQ formula, its *in vivo* therapeutic efficacy, pharmacokinetic profile, bioavailability, and long-term safety in relevant AR models remain to be rigorously validated before clinical translation. Future studies should focus on these aspects to bridge the gap between mechanistic findings and clinical application.

## Conclusion

In conclusion, this study demonstrates a mechanistic framework for YPTQ's action in AR: it concurrently modulates the immune response by inhibiting the TSLP-mediated immunoinflammatory response and promoting the AhR/CYP1A1-mediated epithelial barrier repair. The identification of kaempferol as a key dual-targeting component of YPTQ offers a molecular basis for the formula's integrated “symptom and root cause” treatment strategy. These findings not only elucidate a novel multi-target pharmacological mechanisms of YPTQ but also highlight the AhR and TSLP pathways serve as promising synergistic targets for AR therapy, offering a novel strategic perspective for the application of traditional Chinese medicine in the management of AR.

## Data Availability

The raw data supporting the conclusions of this article will be made available by the authors, without undue reservation.
