# Supplementary material for: Multi-targeted protection of Yuping Tongqiao against allergic rhinitis: suppression of inflammatory response via TSLP signaling and reinforcement of epithelial barrier integrity via AhR signaling
Source: Front Allergy. 2026 May 8;7:1824120. doi: 10.3389/falgy.2026.1824120 (PMC13194122; doi:10.3389/falgy.2026.1824120)
Supplement: Supplementary file 1 [file Table1.docx]

| Component | Latin Name | Medicinal Part | Percentage (Dose of herb / Total dose) |
| --- | --- | --- | --- |
| Huang Qi | *Astragalus membranaceus* (Fisch.) Bunge | Root | 13.5% |
| Bai Zhu | Atractylodes macrocephala Koidz. | Rhizome | 9.0% |
| Fang Feng | Saposhnikovia divaricata（Turcz.）Schischk. | Root | 9.0% |
| Xin Yi | ***Magnolia biondii* Pamp.** | Flower bud | 9.0% |
| Bai Zhi | *Angelica dahurica* | Root | 9.0% |
| Gao Liang Jiang | Alpinia officinarum Hance | Rhizome | 9.0% |
| Qiang Huo | ***Notopterygium incisum* Ting ex H. T. Chang** | Rhizome and root | 9.0% |
| Mu Dan Pi | Paeonia suffruticosa Andr. | Root bark | 9.0% |
| Chan Tui | Cryptotympanaatrata（Fabricius） | Exuviae (shed skin) | 9.0% |
| Wu Mei | Prunus mume（Sieb.）Sieb. et Zucc. | Near-ripe fruit | 9.0% |
| Gan Cao | ***Glycyrrhiza uralensis* Fisch.** | Root and rhizome | 5.4% |
