# Supplementary figures and images for: Multi-targeted protection of Yuping Tongqiao against allergic rhinitis: suppression of inflammatory response via TSLP signaling and reinforcement of epithelial barrier integrity via AhR signaling
Source: Front Allergy. 2026 May 8;7:1824120. doi: 10.3389/falgy.2026.1824120 (PMC13194122; doi:10.3389/falgy.2026.1824120)

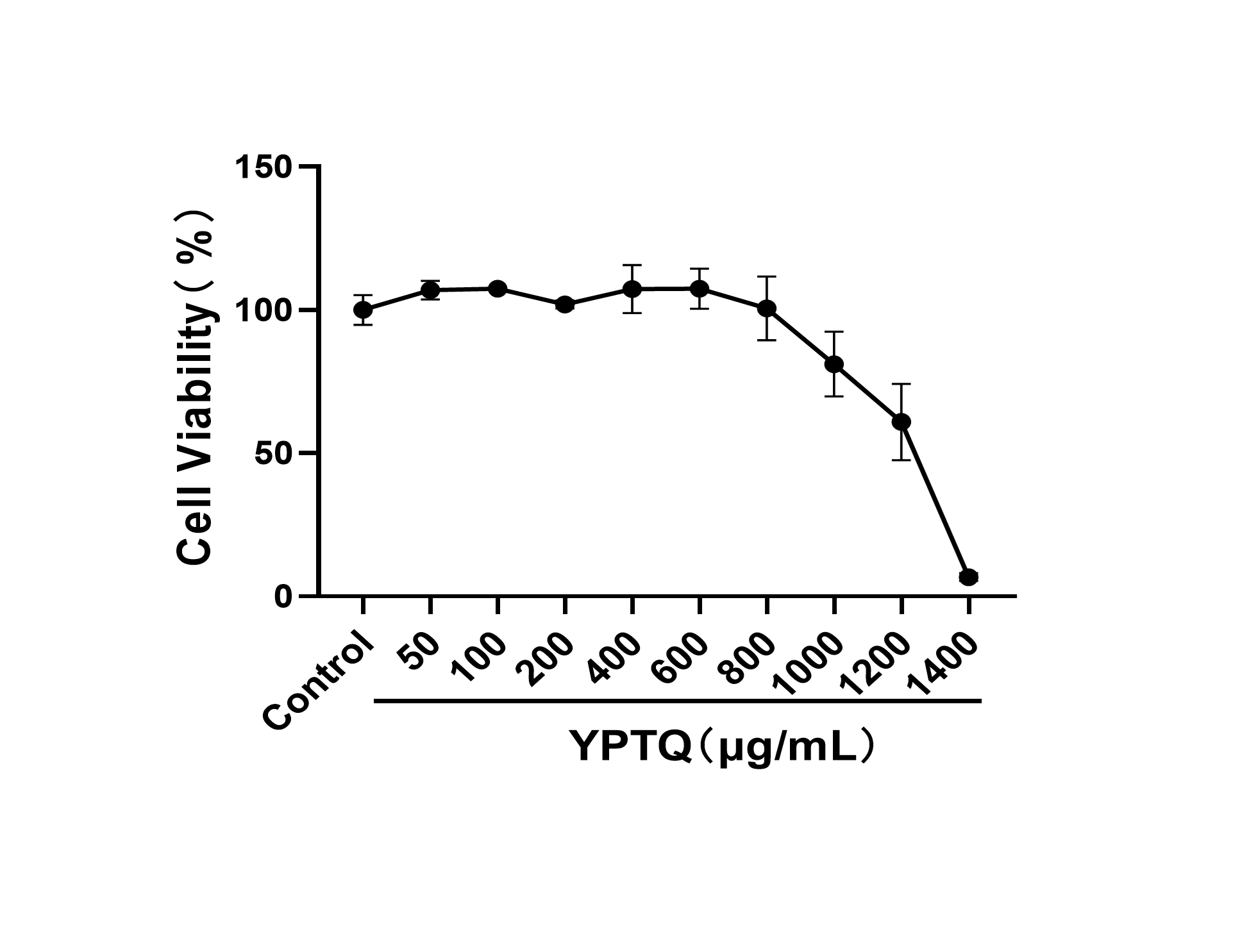

Supplement: Supplementary file 2 [file Image1.tif]
